# The TICAP-Study (titanium clips for appendicular stump closure): A prospective multicentre observational study on appendicular stump closure with an innovative titanium clip

**DOI:** 10.1186/s12893-015-0068-3

**Published:** 2015-07-17

**Authors:** Alexander Rickert, Colin M. Krüger, Norbert Runkel, Andreas Kuthe, Jörg Köninger, Boris Jansen-Winkeln, Carsten N. Gutt, Daniel R. Marcus, Brian Hoey, Moritz N. Wente, Peter Kienle

**Affiliations:** Department of Surgery, University medical centre Mannheim, University of Heidelberg, Theodor-Kutzer-Ufer 1-3, D-68167 Mannheim, Germany; Department of Surgery, Vivantes-Humboldt Klinikum, D-13509 Berlin, Germany; Department of Surgery, Schwarzwald-Baar-Klinikum, D-78052 Villingen-Schwenningen, Germany; Department of Surgery, DRK Krankenhaus Clementinenhaus, D-30161 Hannover, Germany; Department of Surgery, Katharinenhospital, D-70174 Stuttgart, Germany; Department of Surgery, University medical centre Mainz, D-55131 Mainz, Germany; Department of Surgery, Klinikum Memmingen, D-87700 Memmingen, Germany; Marina del Rey Hospital, Marina del Rey, California, CA 90292 USA; General Surgery, St. Luke’s university hospital, Bethlehem, PA 18015 USA; Medical Scientific Affairs, Aesculap AG, D-78532 Tuttlingen, Germany

**Keywords:** Laparoscopic appendectomy, Appendix stump closure, Acute appendicitis

## Abstract

**Background:**

To evaluate the effectiveness and safety of the DS Titanium Ligation Clip for appendicular stump closure in laparoscopic appendectomy.

**Methods:**

Overall, 502 patients undergoing laparoscopic appendectomy were recruited for this observational multicentre study in nine study centres between October 2011 and July 2013. The clip was finally applied in 390 patients. Primary outcome variables were feasibility of the clip, intra-abdominal surgical site (abscesses, stump leakages) and superficial wound infections. Patients were followed 30 days after surgery.

**Results:**

The clip was applicable in nearly 80 % of patients. Reasons for not applying the clip were mainly an inflamed caecum or a too large diameter of the appendix base. Superficial wound infections were found in nine (2.31 %), intra-abdominal abscesses in five (1.28 %), appendicular stump leak in one (0.26 %), and other adverse events in 22 (5.64 %) patients. In total, 12 (3.08 %) patients were re-admitted to hospital for treatment. Seven re-admissions were surgery-related; ten (2.56 %) patients had to be re-operated. One patient died during the course of the study due to persisting peritonitis (mortality 0.26 %).

**Conclusions:**

The results suggest that the DS Titanium Ligation Clip is a safe and effective option in securing the appendicular stump in laparoscopic appendectomy. The complication rates found with the use of the DS-Clip are comparable to the rates in the literature when other methods are used.

**Trial Registration:**

NCT01734837.

## Background

Appendectomy is the most frequent emergency operation in developed countries with about 135.000 operations in Germany every year. A recent survey showed that about 86 % of appendectomies are performed laparoscopically [1]. Laparoscopic appendectomy can be regarded as a well-established surgical technique, with some proven advantages compared to conventional appendectomy (faster recovery, smaller rate of wound infections) [2].

However, there is some debate about the best method for the closure of the appendicular stump. The appendix stump closure plays a key role in preventing infectious complications and is therefore regarded as one of the crucial steps in the operation. Pre-knotted loops, linear staplers and polymeric clips are being used for stump closure. The debate on which is the best method is still on-going [3–5].

A new device for appendicular stump closure is the DS Titanium Ligation Clip (DS-Clip, Aesculap®). It is made of pure titanium, a proven biocompatible implant material that allows for a good adaptation to the tissue as well as a constant and high closing force. The design of the DS-Clip is characterized by two parallel and interconnected shanks. In the resulting space between the shanks, the tissue is compressed during the closing process, providing extra protection against axial displacement. The two shanks additionally prevent clip scissoring, known from single shank clips. The inner surface of the clips have a pyramid-shaped surface imprint which allows the tissue to sink in between the pyramid shapes and thereby, by increasing the contact surface of the tissue, ensuring a strong grip. A latch at the tip of the clip further prevents tissue slippage.

The product has been approved for clinical use (CE-Mark, FDA approval). An initial evaluation in 100 laparoscopic appendectomies showed that the DS-Clip allows for a safe closure of the appendicular stump and that it is easy to handle [6]. The objective of this study was to evaluate the effectiveness of the Aesculap DS Titanium Ligation Clips for appendicular stump closure in laparoscopic appendectomy in real-life clinical routine in a larger number of patients in an international multicentre setting. The effectiveness parameters were applicability/feasibility of the product and surgery related complication rates. This should allow a scientific comparison with other methods described in the literature.

## Methods

### Study design

The study was performed as a multi-centre, prospective, non-controlled, observational study without specification of special treatment protocols. Participating physicians were instructed to continue to treat all patients according to their own best clinical judgement and to submit information on the parameters and outcomes of this treatment to the TICAP database.

The intra-operative and post-operative results of 502 patients with an indication for a laparoscopic appendectomy and an intended appendicular stump closure with the Aesculap DS Titanium Ligation Clips were documented by using an online study database. Patients who met the inclusion criteria and who gave their informed consent to the use of their data were enrolled in the study. The documentation included pre-operative demographic data, intra-operative data, and the outcomes assessed during a follow-up time of 30 days.

### Ethical considerations

The investigational product is approved for use, i.e. bears the CE-mark and has the FDA approval. In this study, the product was used within its indication. Furthermore, there were no additional examinations or interventions performed on the patients. Therefore, this study was not considered to constitute an additional risk for enrolled patients. It was the decision of the treating surgeon if the device was to be used in any patient or not.

An ethical approval was obtained from the appropriate ethics committees of all participating study centres before the enrolment of patients started. Written informed consent was obtained by the investigators at each site from all patients before patient enrolment.

### Inclusion and exclusion criteria

Patients over the age of 16 scheduled for laparoscopic appendectomy (3-port) were recruited preoperatively in the clinical study centres. Approved consent was a prerequisite for inclusion into the study. Other laparoscopic techniques (SILS, NOTES) were exclusion criteria. An intraoperative finding of a severely inflamed caecum was regarded as contraindication for the clip. Perforated appendicitis and a perityphlitic abscess were no exclusion criteria. The decision if the clip could be definitely used was drawn intra-operatively.

### Treatment and treatment allocation

Laparoscopic appendectomy and all pre-operative or concomitant treatments were performed according to local standards. Appendectomy was performed with the standard 3-port approach. The DS-Clip was used according to the instructions for use. Before study start, each centre was informed about the study procedure and trained how to use the device during an initiation visit. The application of one clip on the stump and one clip to close the resected appendix was estimated sufficient and recommended. It was the surgeon’s decision to use an extra clip. The surgeons were asked to estimate the degree of inflammation of the appendix base using a scale from 0 (none) to 3 (severe inflammation). The device-relation of adverse events was judged by the surgeon according to the WHO-UMC Causality Categories (The World Health Organisation’s (WHO) Collaborating Centre for international Drug Monitoring: the Uppsala Monitoring Centre (the UMC)).

### Outcome variables

Primary outcome variables were:

- Applicability/feasibility of the DS-Clip: documentation of the number of successful (= completed) versus non-successful (= clip could not be applied) applications
- Intra-abdominal surgical-site infection
- Superficial wound infection
- Appendicular stump leak (blowout, fistula)

Secondary outcome variables were:

intra- and post-operative complications related to surgery, duration of operation (time from skin incision to skin closure), number of clips used, conversion rate to open surgery, re-operation and re-admission to hospital within the follow-up time of 1 month and duration of hospital stay (day of surgery to discharge).

### Follow-up

As Follow-up, a telephone interview was conducted 30 days after surgery. Adverse events, re-admissions to hospital and necessary medical treatment related to the operation were recorded.

### Statistical analysis

All the data obtained during this study was tabulated and subjected to the standard methods of statistical analysis. Since there was no specific hypothesis to be tested, no specific statistical test methods and no statistically calculated sample size were defined.

However, it was estimated that a minimum patient number of 400 had to be enrolled to allow for a valid comparison with data from the literature. The estimate of the number of patients to be included in the study was based on the frequency of laparoscopic appendectomy associated morbidity as known from scientific publications. The morbidity associated with laparoscopic appendectomy that could possibly be related to stump closure is relatively rare. The incidences for superficial wound infection rates and for intra-abdominal abscesses range from 1 % to 5 %. A pooled analysis of data from randomised studies that mentioned the method of stump closure was performed [2–4, 7, 8].

It was assumed that in approximately 20 % of the cases the clip could actually not be used although intended. In order to compensate for this number a minimum of 500 patients were enrolled in the study to yield a number of approximately 400 patients with the clips actually used.

### Device description

The Aesculap DS Titanium Ligation Clips are made of pure titanium (acc. to ISO 5832–2), a proven biocompatible implant material. The clips are non-ferromagnetic and suitable for use under MRI with fields of up to three Tesla. They are applied with a suitable applier for use in laparoscopic surgery.

**Fig. 1:**
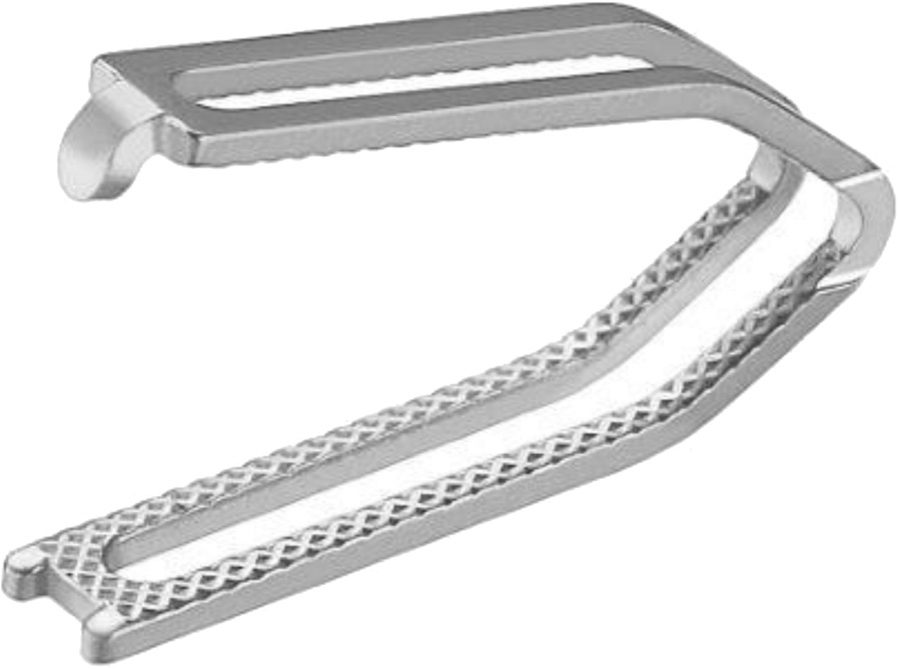
DS-Clip. The Aesculap DS Titanium Ligation Clips are made of pure titanium. They are non-ferromagnetic and suitable for MRI. The design of the DS-Clip is characterized by two parallel and interconnected shanks, which prevent axial displacement and clip scissoring. The pyramid-shaped inner surface increases the contact surface of the tissue, ensuring a strong grip

The Aesculap DS Titanium Ligature Clips devices are intended for use in endoscopic and open surgery for the ligation of blood vessels and hollow organs whenever clips are indicated (Fig. 1).

## Results

### Demographic data

A total of 502 patients were enrolled into the TICAP Study between October 2011 and July 2013. Patients were recruited in nine study centres (Table 1). The study centres 008 and 009 had less time to enrol patients (0,43 and 0,68 years, respectively compared to 1,76 years). This means an average patient enrolment of 3 and 2 patients (2 US study centres) patient per months compared to 3 patients (3 German study centres), 4 patients (2 German study centres), 5 patients (1 German study centre) and 6 patients per month (1 German study centre).

**Table 1:** Patient recruitment per site

Study site		Patients recruited per study site (per month)	Number/% of appendectomies performed with DS-Clip	
No.	Name			
001	Universitätsklinikum Mannheim	58 (3)	48	82.8 %
002	Katharinenhospital Stuttgart	61(3)	43	70.5 %
003	Vivantes Humboldt-Klinikum Berlin	75 (4)	61	81.3 %
004	Klinikum Memmingen	50 (3)	33	66.0 %
005	DRK Krankenhaus Clementinen Hannover	74 (4)	66	89.2 %
006	Schwarzwald-Baar Klinikum Villingen-Schwenningen	91 (5)	69	75.8 %
008	Marina del Rey Hospital Marina del Rey, California, USA	20 (2)	20	100.0 %
009	St. Luke’s University Hospital Bethlehem, Pennsylvania, USA	13 (3)	13	100.0 %
010	Universitätsklinikum Mainz	60 (6)	37	61.7 %
	TOTAL	502	390	77.7 %

**Fig. 2:**
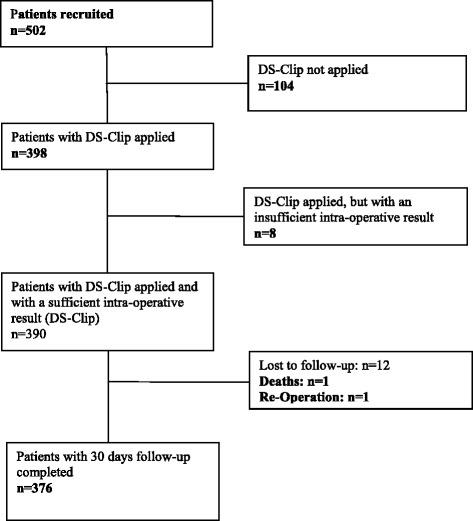
Flow of patients. In all 502 recruited patients, the intended treatment was laparoscopic appendectomy with the DS-Clips to be used for the ligation of the appendix (recruited patient). All patients actually treated with the DS-Clip were considered in the DS-Clipgroup, *n* = 390

In all 502 recruited patients, the intended treatment was laparoscopic appendectomy with the DS-Clips to be used for the ligation of the appendix (recruited patient). All patients actually treated with the DS-Clip were considered in the DS-Clipgroup, n = 390. The flow of patients is shown in Fig. 2, the demographic data for all patients in Table 2.

**Table 2:** Demographic data of recruited patients and of patients treated with DS-Clip and showed sufficient intra-operative results (DS-Clip^a^)

	Recruited Patients, *n* = 502		DS-Clip^a^, *n* = 390	
	Mean	SD	Mean	SD
Age, years	35.33	16.44	34.14	15.79
BMI, kg/m^2^	25.25	4.85	25.03	4.77
	n	%	n	%
Female	246	49.00	199	51.03
Male	256	51.00	191	48.97
ASA I	258	51.39	208	53.33
ASA II	194	38.65	142	36.41
ASA III	48	9.56	39	10.00
ASA IV	2	0.40	1	0.26

*BMI* body mass index, *ASA* American Society of anaesthesiologists, *SD* standard deviation

^a^Patients treated with DS-Clip and showed sufficient intra-operative results according to Fig. 2

### Intra-operative findings

In the group of patients where the DS-Clip was used, the diameter of the appendix base was estimated to be 8.16 mm on average (SD 2.43; n = 390; range 3 – 20 mm).

In the DS-Clipgroup intra-operative macroscopic assessment showed acute appendicitis in 312 (80.00 %) patients, of whom 32 (8.21 %) patients had a perforated appendicitis. In 78 (20.00 %) patients, appendicitis was macroscopically not visible. In 15 (3.85 %) of these patients a different pathology was identified as the cause of symptoms.

Histological examination showed acute appendicitis in 347 (89.00 %) of patients (Table 3).

**Table 3:** Intraoperative and histological findings of patients treated with DS-Clip (*n* = 390)

	n	%
Macroscopic state of appendix		
Acute appendicitis	312	80.00
Not perforated	280	71.79
Perforated	32	8.21
No appendicitis	78	20.00
No other pathology	63	16.15
Other pathology	15	3.85
Inflammation of appendix base		
None	198	50.77
Slight	129	33.08
Moderate	51	13.08
Severe	12	3.08
Histological assessment of appendix		
Appendicitis	347	89
No appendicitis	43	11
Size of appendix base		
Mean ± SD, mm	8.16 ± 2.43	
range, mm	3.00 - 20.00	

Inflammation of the appendix base as judged by the surgeon, scale 0–3: 0 none, 1 slight, 2 moderate 3 severe

*SD* standard deviation

### Applicability of the DS-Clip

In 104 patients (20.72 %), the DS-Clip was not used, although intended. The reasons for not using the DS-Clip and the frequencies are listed in Table 4. In another group of eight patients, the DS-Clip was applied but after application, it was realised that the stump closure was not sufficient, because of a too large base or inflamed caecum. Thus, another method for the closure was used during the same surgical procedure. In 12 patients (3.08 %) the DS-Clip was applied, though the appendix base was estimated to be severely inflamed. None of these patients developed an intra-abdominal abscess. In 6 patients (1.54 %) with applied DS-Clip, the appendix base was estimated to be 15 mm or larger. No adverse event occurred in these patients.

**Table 4:** Reasons for not primarily using the DS-Clip or for using additional closure methods

DS-Clip not primarily applied	n	%
Base of appendix too large	44	8.76
Caecum inflamed	41	8.17
Conversion to open surgery	9	1.79
No appendicitis	5	1.00
Lack of clips/applicator in the OR	5	1.00
Total	104	20.72
Additional closure method necessary	n	%
Base of appendix to large	6	1.20
Caecum inflamed	2	0.40
Total	8	1.59

*OR* operation room

There were 9 conversions (1.79 %); 8 (1.59 %) due to perforated appendicitis, 1 (0.19 %) due to adhesions.

In total, there were 112 (22.31 %) patients where the DS-Clip could not to be used for stump closure.

Overall, the clips were actually used to close the appendicular stump in 77.69 % of cases (390 of 502). In 93 patients (18.53 %), the clips could not be used because of reasons related to the clip size and to the product specifications: 50 (9.96 %) patients had an appendix basis too large for the DS-Clip and in 43 (8.57 %) patients the caecum was involved, which is a contra-indication for the DS-Clip. Thus, at least in theory the ratio of patients where the DS-Clip would have been applicable - in terms of the clip indication and its size – was 81.47 % (409 of 502).

One to four clips were used on the appendix. In most cases (64 %), two clips were used.

Additional clips were applied in 112 (28.72 %) patients on other sites (appendicular artery, Table 5).

**Table 5:** Number of clips used on the appendix and on other sites (appendicular artery)

	n	%
Number of clips used on appendix		
One	60	15.38
Two	250	64.10
Three	75	19.23
Four	5	1.28
Number of clips used on other sites		
One	80	20.51
Two	31	7.95
Four	1	0.26
Total	112	28.72

### Duration of surgery and length of hospital stay

The duration of surgery from incision to closure of the skin was on average 45 min (SD 21.22). Mean length of hospital stay was 3.77 days (SD 2.05).

The 30 days follow-up examination (telephone interview) was available for 376 (96.4 %) patients. The interview was conducted on average 36 days after surgery (SD 21.74).

### Complications

There were no adverse device effects related to the DS-Clip. Three adverse events were judged as probably related to the DS-Clip: one intra-abdominal abscess, one intra-operative bleeding from a clipped artery, and one appendicular stump leak. In 31 of 390 (7.95 %) patients overall 37 adverse events were reported – 22 (59.46 %) of them were classified as serious.

Superficial wound infections were found in 9 (2.31 %) patients, intra-abdominal abscesses in 5 (1.28 %) patients, appendicular stump leak in one (0.26 %) patient, and other adverse events in 22 (5.64 %) of patients (Table 6).

**Table 6:** Complications and adverse events of patients treated successfully with the DS-Clip (*n* = 390)

	n	%
Superficial wound infection	9	2.31
Intra-abdominal abscess	5	1.28
Appendicular stump leak	1	0.26
Persistent pain	7	1.79
Haematoma wound	2	0.51
Abdominal bleeding	3	0.77
Ongoing Peritonitis	1	0.26
Other^a^	9	2.31
Re-operations	11	2.82
During initial stay	8	2.05
Upon re-admission	3	0.77
Re-admissions to hospital	12	3.08
Causality of adverse events^b^		
Not assessable	2	0.51
Unlikely	32	8.21
Possible	3	0.77
Probable	0	0
Certain	0	0

^a^Other adverse events: Enteritis, persistent elevated inflammatory parameters, sepsis with MRSA, persistent diarrhoea, persistent numbness of the wound, intra-abdominal fluid collection, constipation, malfunction of monopolar device and injury of abdominal structure, clip fell of applicator

^b^Causality of adverse events was judged by the surgeon according to the WHO-UMC Causality Categories (The World Health Organisation’s (WHO) Collaborating Centre for international Drug Monitoring: the Uppsala Monitoring Centre (the UMC))

In total, 12 (3.08 %) patients were readmitted to hospital for treatment. Reasons for re-admission were persistent postoperative pain (5), intra-abdominal abscess (4), persistent elevated inflammatory parameters (1), enteritis (1), wound infection (1), and corpus luteum cyst (1).

In 10 (2.56 %) patients 11 re-operations had to be done, of which eight were performed during the initial hospital stay and three after re-admission to hospital. Reasons for surgical re-interventions were: intra-abdominal abscess (4), intra-abdominal fluid collection (1); intra-abdominal haematoma (2), lower abdominal pain with rising CRP and WBC (1); bleeding trocar access site (1); ongoing peritonitis without stump leak (1); MRSA sepsis (1); appendicular stump leak (1). One patient was re-operated two times, because of ongoing peritonitis with sepsis caused by MRSA (Table 6).

### Mortality

One patient died during the course of the study (mortality 0.26 %). The male patient, age 36 years, ASA grade 2, was re-operated two times due to on-going peritonitis. The appendicular stump showed to be intact. The cause of death was MRSA sepsis with pneumonia. The death was not related to the DS-Clip or to the surgery.

## Discussion

Over the past decade, laparoscopic appendectomy became the preferred technique for the treatment of acute appendicitis. Recent studies show that up to 86 % of appendectomies are nowadays performed laparoscopically [1, 9]. Several advantages of the laparoscopic approach such as shorter hospital stay, faster recovery and lower wound infection rate were shown in many studies and meta-analysis. On the other hand a slightly increased intra-abdominal abscess rate was found for complicated appendicitis in comparison to the open approach [2, 8].

Appendicular stump closure is an essential part in avoiding infectious complications. Endostaplers, endoloops and endoclips are used for this procedure in acute appendicitis. All devices can be used for adequate stump closure with every method having its benefits and disadvantages. The available literature is heterogeneous and a general recommendation for either one of the methods cannot be given.

Several studies compared staplers to endoloops. In two reviews the routine use of endostaplers is favoured, especially in case of an inflamed appendix base, because a lower complication rate was found compared to endoloops [3, 5]. In contrast, another review, compiling five RCTs with 622 patients, concludes superiority of endoloops because of similar complication rates compared to staplers but much lower costs [4]. However, the use of endoloops resulted in a longer operation time in these studies, which therefore also leads to higher costs. An additional disadvantage of the endoloops is that there is a certain amount of experience required for the placement and tightening of the loop around the appendix base [10].

The third group of devices, the endoclips are less commonly used and have been less well investigated, although the use of clips for appendicular stump closure was described more than 20 years ago [11]. The use of endoclips is technically limited, depending on the severity of the inflammation and the diameter of the appendix base. Small cohort studies using metal endoclips showed that these clips can be used safely for appendicular stump closure in selected cases [6, 12]. Two recent small randomised trials found comparable complication rates and a shorter operation time when metal clips were used instead of intracorporal knotting techniques [13, 14]. Studies that compare metal endoclips to staplers or endoloops have not yet been published. Polymeric none-metal clips have been used in several studies, and resulted in shorter operation times, reduced costs and comparable complication rates [15–19].

A recent review stated, it should be up to the decision of the surgeon which method of stump closure he uses, since high quality evidence for the superiority of either one of the described techniques is lacking [5]. First of all the optimal device should result in a safe stump closure with a low rate of complications and offer a maximum of safety for the patients. Furthermore, it should be cheap and easy to handle.

In this multicentre study the safety and effectiveness of the titanium DS-Clip for appendicular stump closure in laparoscopic appendectomy was investigated. In nine study centres throughout Germany and in the United States, 502 patients were enrolled.

Hospitals of different size and supply levels, ranging from country hospital to university hospital in two countries were among the study centres. The surgery was performed by surgeons with different levels of expertise in laparoscopic surgery (minimum standard: supervision of a consultant surgeon). It was thought that this would meet the daily clinical situation quite well. For the same reason as few as possible specifications were made and the appendectomy procedure was not further standardized.

The demographic data of the study population was comparable to that of a large quality assurance study on 17732 patients in Germany from 2013 [1]. Therefore, it can be assumed that the screened study population in the presented study is representative for the population of patients undergoing laparoscopic appendectomy.

The negative appendectomy rate of 11 % is concordant with the literature where negative appendectomy rates between 10 and 15 % are described [20].

The DS-Clip could not be applied in 22 % of patients. The reasons for not using the clips that were directly related to clip features were an appendix base too large for the clip size (in 10 %) and a severely inflamed appendix base (in 9 %). The other reasons for not using the clip were not related to clip features. Thus, it can be stated that in a typical population of appendectomy patients the clip is applicable in around 80 % of patients.

These limitations, severely inflamed or too large appendix base, are also of relevance when using other devices such as endoloops or polymeric clips. Under these circumstances, only the use of a stapler results in a safe stump closure as even a severely inflamed appendix base can be removed by stapling with larger or multiple cartridges through the healthy caecal wall.

However, the used titanium clip could also safely be applied in a substantial number of patients with an inflamed appendix base in this study as the appendix base was estimated as at least slightly inflamed in half of the patients. Recent non-randomised studies concluded that the polymeric hem-o-lok clips could be used in cases with an appendix base up to 10 mm, endoloops in cases up to 15 mm [17, 18]. The titanium clip was safely applied for closure of the appendix bases with a diameter of up to 20 mm.

In almost two thirds of patients, two clips were used at the appendix site. In 15 %, the surgeons decided to use only one clip. In this case, it is advisable to transfer the appendix immediately into a retrieval bag to avoid contamination. In 20 % of cases, three clips were used with an extra “safety” clip at the appendix base, though it has to be mentioned that one clip at the base is basically regarded sufficient.

Surgeons decided to use the clip for ligating the appendicular artery or the mesoappendix in 30 % of cases. The DS-Clip is safe and efficient for ligation of appendicular arteries, as no postoperative bleeding occurred in these cases. Monopolar cauterization, staplers and clips have been shown to be equally effective in obtaining vascular control of the appendicular artery [21].

Safety and efficacy are interrelated parameters in this study. The effectiveness of the device is mainly reflected by the absence of complications. Typical major complications of appendectomy that generally need interventional or surgical treatment are intra-abdominal abscesses (IAA) and appendicular stump leaks. Usually it is difficult to distinguish stump leakage from an IAA in the right lower abdomen at the former appendix site even if the patient is re-operated. As IAAs are nowadays frequently treated by interventional drainage the rate of stump leakages might be underestimated. In the literature mostly only, the rate of intra-abdominal abscesses is mentioned or both complications are summarized under intra-abdominal surgical site infections. A Cochrane systematic review from 2010 found a higher rate of IAAs after laparoscopic appendectomy compared to open appendectomy, while the rate of superficial wound infections was significantly higher in open appendectomy. In this review an IAA-rate of 1.8 % and a superficial wound infection rate of 3.4 % were found after laparoscopic appendectomy [2]. More recent reviews and large single-centre studies also showed IAA-rates between 1.3 % and 2.2 % and wound infection rates up to 4 % after laparoscopic appendectomy [7, 8, 22, 23]. These complication rates are comparable to those found in this study. However, in these reviews and studies, the appendix stump closure was mostly achieved by the use of linear staplers or endoloops, partly these two methods were alternately used in one study. A comparison between the different methods with regard to complications was not performed. A pooled data analysis of the studies in this review which reported the use of either one method showed IAA-rates of 2.7 % for Endostaplers and 2.5 % for Endoloops and wound infection rates of 5.1 % (Staplers), respectively 3.1 % (Endoloops).

Meta-Analysis comparing staplers with endoloops for appendix stump closure found no significant differences for IAA-rates (4.7 % for staplers versus 5.7 % for endoloops), though the number of patients in the included studies was small and the quality of the studies was poor [3, 4]. In a retrospective analysis on 6468 patients the IAA-rate was 1.1 % when staplers were used, which was not significantly lower than an IAA-rate of 1.6 % when endoloops were used [10]. For the use of metal clips for appendix stump closure, only few studies are available. The rate of IAA in these studies ranges from 1 % to 1.6 %. The wound infection rate ranges also from 1 % to 1.6 % [12, 13].

In summary, the rates for intra-abdominal and superficial surgical site infections in this study are comparable with the complication rates published in literature for different methods of appendix stump closure.

Large cohort studies reported of shorter operation times, when endostaplers where used (51.7-58 min) compared to endoloops (53.4-60 min). The operation time in this study was 45 min, which is comparable to other studies that examined stump closure with endoclips (46.3-64.9 min) [10, 13, 18, 24].

The length of hospital stay varies widely, depending much on, in which country the study was performed. This is also remarkable in our study. The centres in the US had a shorter hospital stay; most patients were discharged the day after surgery. Recent studies found a median hospital stay from 2 to 5.9 days with no significant differences between the different methods of appendix stump closure [2, 10, 24].

A recently published large German quality assurance study (more than 17.000 patients) found a conversion rate of 6.1 % in laparoscopic appendectomy. The low conversion rate of 1.79 % found in our study is most likely due to the exclusion of severely inflamed appendix with involvement of the caecum. Nevertheless the rates of perforated appendicitis and the basic patient data (age, BMI, ASA) are comparable between the two studies. The rates for intra-abdominal abscesses (1 %) and wound infections (3 %) were comparable to our study. In 86 % of cases a stapler was used to close the appendix stump [25].

Studies that examine the use of clips for appendix stump closure found conversion rates between 1 and 4.9 % [12, 18].

The use of clips instead of staplers finally results in reduced costs, when compared to the use of staplers, which is currently the most common method of appendix stump closure. The price of a set of 4 DS-Clips is about 80€ (Endostapler ~ €300, two endoloops ~ €20). When regarding the comparatively low costs of endoloops, it must be considered that the operation time with endoloops seems to be longer and so additional costs apply.

It also has to be considered that adverse events cause costs through reoperations, reinterventions, medical treatment and prolonged hospital stay.

Data is not consistent concerning the different stump closure methods. A recent study compared routine use of staplers with selective use of staplers or endoloops (staplers were used only in cases with inflamed or perforated appendix base). A selective use of staplers did not lead to higher complication rates but led to reduced costs (4.1 million euros annually) [24].

A retrospective study from 2006 compared staplers to endoloops and found a higher readmission rate in patients treated with endoloops, which rendered staplers more cost effective when regarding the total costs [10].

The limitation of the study is that there was no randomised control group with an alternative method for stump closure. This was because the complication rate in laparoscopic appendectomy is low and a very large number of patients would have to be tested to reach statistical power with this endpoint. There is also a bias as more severe cases (severely inflamed appendix with involvement of the cecum, appendix base to large) were not treated with the clip and therefore excluded. This might explain the different rates of clip application throughout the centres.

## Conclusion

The DS-Clip is a safe and effective device for closing the appendix base in laparoscopic appendectomy. The clip was applied in large number of patients in university and country hospitals. The DS-Clip shows a comparable complication rate to other used methods. We think the DS-Clip is suitable as a standard tool for laparoscopic appendectomy in hospitals of different supply levels. There are some limitations for the clip application in case of severely inflamed or wide appendix base. Though a group of patients was excluded, when these conditions were met, nevertheless in a group of patients the clip was applied, even when the appendix base was estimated severely inflamed or extremely enlarged. In these cases the surgeon’s evaluation of the stump closure plays a key role.

## Abbreviations

ASA: American Society of Anaesthesiologists
BMI: Body mass index
CRP: C-reactive protein
IAA: Intra-abdominal abscess
MRSA: Methicillin-resistant staphylococcus aureus
RCT: Randomised controlled trial
SD: Standard deviation
TICAP: Titanium clips for appendicular stump closure
WBC: White Blood Count
